# Implantation Metastasis Following a Biopsy of Thalamic Germinoma: A Case Report

**DOI:** 10.7759/cureus.74377

**Published:** 2024-11-25

**Authors:** Llarx Saundt Yu, Oliver Ryan Malilay, Joseph Erroll V Navarro

**Affiliations:** 1 Neurosurgery, Jose R. Reyes Memorial Medical Center, Manila, PHL

**Keywords:** complication, germinoma, implantation metastasis, seeding, stereotactic biopsy, thalamic tumor

## Abstract

Germ cell tumors (GCTs) commonly develop in the pineal and suprasellar regions, with the most common GCTs being germinomas. In this report, a 22-year-old male presented with progressive right-sided weakness, and his imaging was consistent with a left thalamic high-grade glioma. A stereotactic biopsy was performed, revealing a germinoma, but radiation therapy was not done, and the patient was lost to follow-up. He was readmitted due to ulcerative tumor growth at the previous skin incision, and imaging revealed tumor progression with implantation metastasis along the track of the previous biopsy. The scalp lesion and tumor through the burr hole were excised, and histopathologic studies confirmed that it was a proliferation of the previous germinoma. He was discharged well but was unfortunately unable to follow up at the clinic. Thalamic germinomas are difficult to diagnose and treat because of their rarity. Here, we underscore the importance of careful patient selection, precise biopsy techniques, thorough postoperative management, and close radiographic surveillance to minimize the risk of implantation metastasis and promptly treat it if it occurs.

## Introduction

Primary tumors arising from the thalamus comprise only 1% of adult brain tumors and 2%-5% of pediatric brain tumors. For both populations, the most common type of thalamic tumor is low-grade gliomas; other, rarer histopathologies reported include ependymomas, primitive neuroectodermal tumors, gangliogliomas, and ganglioneurocytomas [1]. As for germ cell tumors (GCTs), the majority are present before the age of 20 (90%), and most are found in the pineal (40%-60%) or suprasellar (30%-40%) regions. On histologic classification, pure germinomas account for more than 50% of GCTs, and mixed tumors and teratomas account for 10%-20% each [1].

The combination of germinoma presenting in the thalamus is unusual, with limited data [2-4]. With varying reports in small case series, germinomas arising in the thalamus and basal ganglia range from 4% to 20% of all intracranial germinomas. These commonly occur in adolescents ages 7-20 years, with male predominance [3,5,6]. The treatment program for this case was challenging due to the atypical presentation and radiologic appearance, pathologic findings, and various societal and economic factors. Adding to this challenge is implantation metastasis from the primary tumor. Brain metastases are the most common tumor of the central nervous system (CNS), but there are few reports on local seeding from a biopsy procedure. Implantation metastasis, or tumor spread along the needle track of stereotactic biopsy, is a very rare complication, reported only in cases of glioblastoma, astrocytoma, pineal tumors, and fibrous histiocytoma [7-12]. To the best of our knowledge, this is the first report on implantation metastasis along the biopsy track from stereotactic biopsy of a thalamic germinoma. Here, we report its signs and symptoms, diagnosis and management, course, and challenges.

## Case presentation

A 22-year-old male presented with a progressive right-sided weakness for two years. His past medical and family medical history were unremarkable. Upon neurologic examination, he was awake, oriented, and able to follow commands, but had slowed mentation. He experienced diplopia and had right lateral rectus palsy and right hemiplegia.

**Figure 1:**
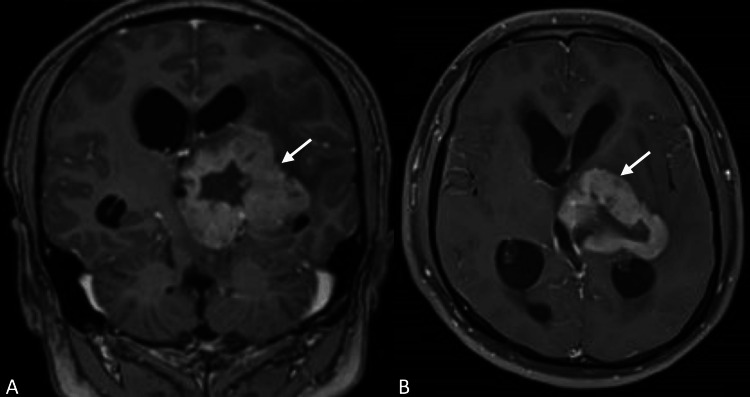
Preoperative T1-weighted contrast MRI (A) Coronal and (B) axial views with enhancing tumor at the left thalamus (arrows) MRI, magnetic resonance imaging

A magnetic resonance image (MRI) obtained revealed a 4.8 cm x 5.4 cm tumor at the left thalamus, with defined margins and heterogeneous contrast enhancement (Figure 1). Under the impression of a probable high-grade glioma, a stereotactic biopsy was performed through the left Kocher’s point, taking samples from four quadrants of the target. This was immediately followed by a contralateral ventriculoperitoneal shunt insertion.

**Figure 2:**
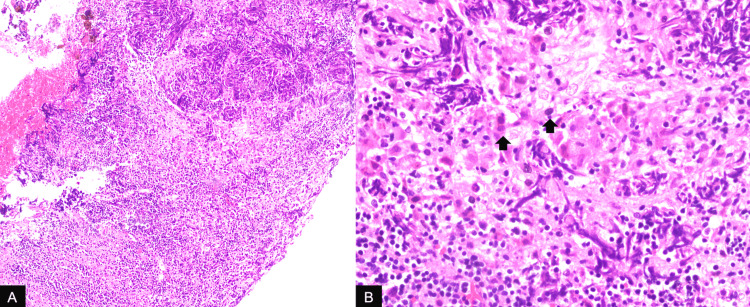
Representative microscopic sections of the left thalamic mass on hematoxylin & eosin (H&E) stain (A) Round cells in sheets and clusters separated by fibrous septa at 100x; and (B) high-power image shows the round cells (as pointed by black arrows) with enlarged, mildly pleomorphic nuclei, prominent nucleoli, and ample eosinophilic cytoplasm, surrounded by lymphocytic infiltrates at 400x

**Figure 3:**
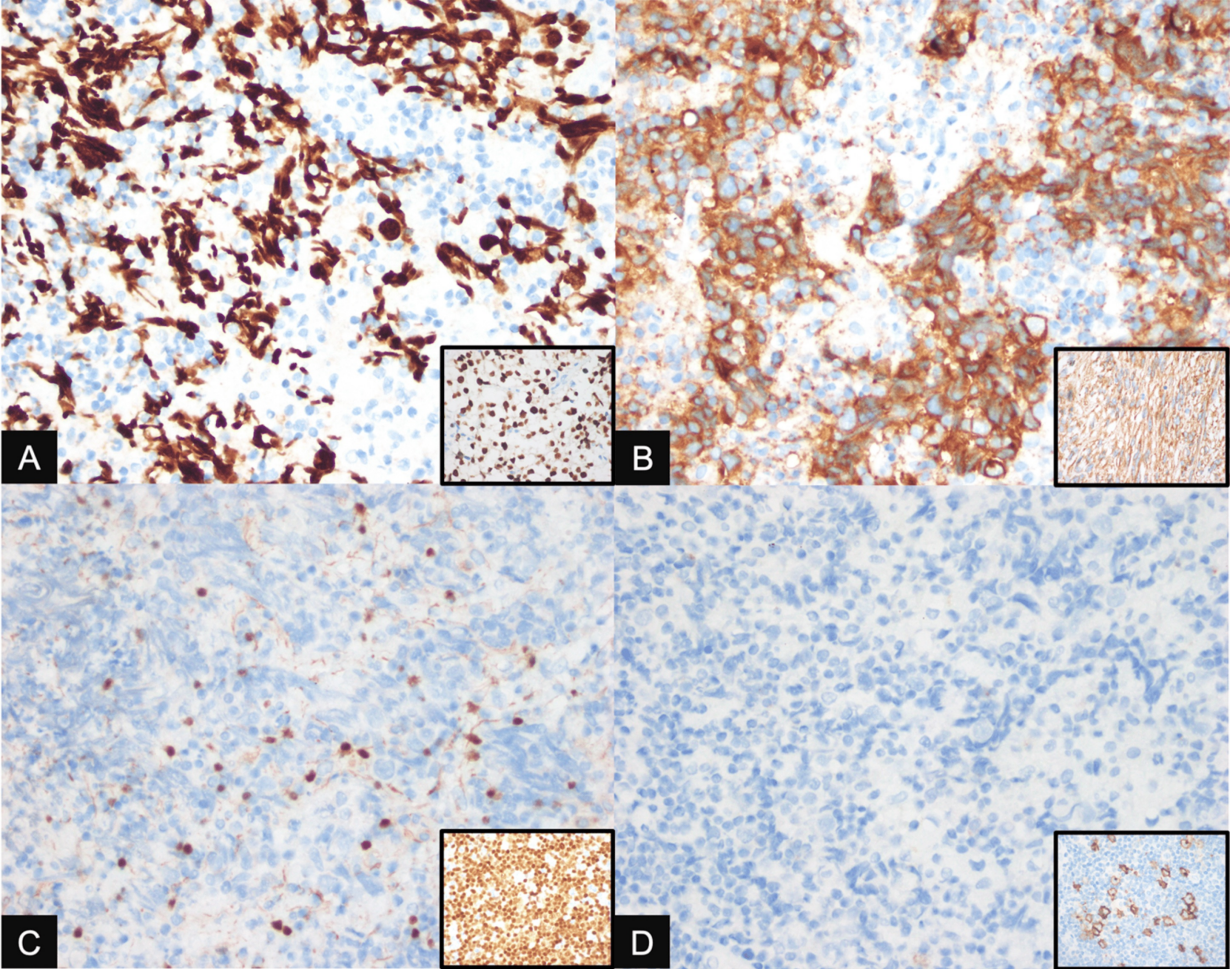
Immunohistochemistry stains on Horseradish peroxidase method at 400x with the respective positive controls (inset) (A) SALL4, (B) CD117, (C) Pax5, (D) CD30

The specimen was submitted for routine histopathologic analysis and primarily showed morphologic features of a round cell malignancy, with considerations of GCT versus Hodgkin lymphoma (Figure 2). Immunohistochemistry studies were performed for further subtyping and revealed positive expression for SALL4 and CD117, with negative expression for Pax5 and CD30 in the round cells. Pax5 expression highlights the presence of a few scattered reactive B lymphocytes (Figure 3). In the end, the immunohistomorphologic profile is compatible with a germinoma. Adjuvant radiotherapy was part of the planned treatment, but, due to undisclosed circumstances, they were lost to follow-up.

Two months later, he developed a progressively enlarging mass under the left frontal post-operative site, which developed a nonhealing, bleeding ulceration. On admission, he had a fungating mass eroding the skin and producing serous discharge at the previous biopsy incision site. He was awake and able to name himself, followed by prodding, with slowed responses, and with a right lateral rectus palsy and right hemiparesis.

**Figure 4:**
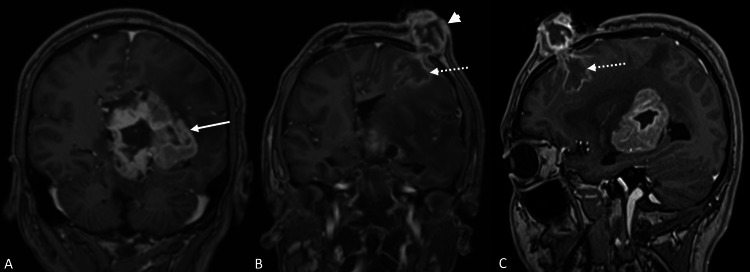
Postoperative T1-weighted contrast MRI taken two months after stereotactic biopsy and VPS (A) Tumor growth (solid arrow), with new growth along the biopsy track (broken arrows) continuous with the scalp (arrowhead) on (B) coronal and (C) sagittal views MRI, magnetic resonance imaging; VPS, ventriculoperitoneal shunt

A repeat cranial MRI showed growth of the tumor to 4.8 cm x 6.4 cm, with tumor extension following the biopsy track at the left frontal parenchyma, to the burr hole, and to the scalp (Figure 4). There was no evidence of metastases on spinal MRI. The impression was implantation metastasis of the GCT extending to the scalp. To repair the skin and obtain tissue confirmation, he underwent sampling of the tumor at the burr hole and excision of the scalp mass, with closure using a rotational flap and split-thickness skin graft. The postoperative course was unremarkable, and he was discharged with a schedule for MRI and radiation therapy. Upon histologic analysis, the tissue taken from the burr site was germinoma, but the scalp tissue was inflammatory and granulation tissue, with no evidence of malignancy. He was discharged with plans for adjuvant therapy, but despite constant advice and communication, he was again lost to follow-up.

## Discussion

Germinomas located in the thalamus and basal ganglia, otherwise called ectopic germinoma, are reported with a higher incidence in the Asian population and usually occur in the second decade of life [5,6]. Its unusual presentation and rarity make it challenging for the clinician. In one report, intracranial germinomas caused granulomatous reactions and inflammatory cerebrospinal fluid (CSF), leading to inaccurate diagnosis and management [13]. In another report, even with a biopsy done, the consideration was high-grade glioma versus lymphoma; the histopathologic diagnosis was inconclusive until the patient underwent tumor resection, and finally, the surgical specimen was examined [4]. This then entails high clinical suspicion for germinoma in the thalamus in this population.

GCTs may have a pattern of findings on imaging. On CT, they have been described as typically hyperdense, and they are hypointense to isointense on both T1- and T2-weighted MRI with contrast enhancement [1]. These features may represent GCTs in general or primary CNS lymphoma, but they should guide the clinician to a probable GCT or nongerminomatous germ cell tumor (NGGCT).

Including GCTs in the differential diagnoses leads to testing for levels of alpha-fetoprotein (AFP) and beta-human chorionic gonadotropin (β-hCG). Following positive results for both β-hCG (>50-100 IU/L) and AFP (>5-10 ng/mL) in serum or CSF, histologic confirmation is not needed for the diagnosis of an NGGCT. Observed in some germinomas, however, is a low-level expression of β-hCG (50-100 IU). On the other hand, in a pure germinoma, AFP is not elevated. Germinomas are highly responsive to chemotherapy and radiation, obviating the need for surgical debulking [1].

When inconclusive, tissue sampling is warranted. Stereotactic biopsy is one way to ascertain histology and is generally safe, but complications can occur. Documented complications include bleeding, infection, new neurological deficits, and seizures. According to a study by Orringer et al. (2012), the overall complication rate for stereotactic brain biopsy was reported to be around 10%, with the most common complications being hemorrhage and transient neurological deficits [14-16].

In addition to the aforementioned complications, there is a risk of autotransplantation or implantation metastasis to the biopsy track site. This occurs when tumor cells from the biopsy track are inadvertently spread during the procedure, leading to the development of new tumor growths along the biopsy path. To minimize the risk of implantation metastasis, several meticulous techniques can be employed during stereotactic brain biopsy. These include limiting the number of biopsy tracks, using non-cutting biopsy needles, ensuring adequate tissue sampling, immediately sealing the biopsy track, performing postoperative imaging, and maintaining close follow-up [15,16].

Several studies have investigated the risk factors and incidence of local recurrence or implantation metastasis after brain tumor biopsy. These studies typically focus on factors such as tumor type, location, biopsy technique, and postoperative management. One notable study by Chang et al. (2008) investigated the risk factors for local recurrence after stereotactic biopsy of gliomas. The study found that factors such as higher tumor grade, incomplete resection, and younger patient age were associated with an increased risk of local recurrence [17]. Another study by Carnevale et al. (2022) focused on stereotactic biopsy of brain metastases, where they found that 6 out of 25 patients developed new metastasis along the track, with a median of five months post-procedure [18]. Like brain metastases, and depending on the histopathologic diagnosis, these were treated with observation or surveillance, systemic therapy, stereotactic radiosurgery, resection, and/or reirradiation [16,18].

Overall, while there may not be a specific threshold for the number of cells needed for implantation metastasis following stereotactic biopsy, present studies underscore the importance of careful patient selection, precise biopsy techniques, thorough postoperative management, and close radiographic surveillance to minimize the risk of implantation metastasis and diagnose its occurrence for prompt management.

## Conclusions

In summary, ectopic germinomas in the thalamus and basal ganglia present significant diagnostic and clinical challenges due to their rarity and atypical presentation, often leading to misdiagnosis and complex management. Differential diagnosis and consideration of GCTs, with clinical findings and imaging, is important, as this may have been confirmed with CSF and blood levels of AFP and β-hCG, without the need for invasive procedures such as biopsy.

High clinical suspicion, appropriate use of tumor markers, and precise diagnostic techniques, such as stereotactic biopsy, are essential for an accurate diagnosis. However, clinicians must be aware of potential rare complications, such as the risk of implantation metastasis. Effective management strategies, including chemotherapy and radiation, along with vigilant postoperative care, are crucial. This underscores the necessity of comprehensive and meticulous approaches in handling these rare intracranial tumors, as well as the challenges posed by patient loss to follow-up. In young patients with potentially curable tumors, maintaining compliance with follow-up protocols is essential to ensure optimal outcomes. This case serves as a reminder of the importance of patient and family education regarding the long-term management of their condition and the need for robust follow-up protocols to avoid treatment gaps.
